# Associative Agreement as a Predictor of Naming Ability in Alzheimer's Disease: A Case for the Semantic Nature of Associative Links

**DOI:** 10.3389/fnbeh.2017.00261

**Published:** 2018-01-09

**Authors:** Gian Daniele Zannino, Roberta Perri, Alice Teghil, Carlo Caltagirone, Giovanni A. Carlesimo

**Affiliations:** ^1^Laboratory of Clinical and Behavioral Neurology, IRCCS Santa Lucia Foundation, Rome, Italy; ^2^Department of Psychology, University of Rome La Sapienza, Rome, Italy; ^3^Department of Neurology, University of Rome Tor Vergata, Rome, Italy

**Keywords:** semantic memory impairment, semantic memory models, Alzheimer's disease, free association, semantic priming

## Abstract

We aimed to address the long-standing issue of the nature of the relationships that link a cue word to words associated with it. In keeping with a recently proposed neuropsychological model of semantic memory (Zannino et al., 2015), we provide support for the hypothesis that associative links are semantic in nature and not lexical. In support of this hypothesis, we demonstrate a relationship in healthy subjects between the probability of producing word X in response to cue word Y in a free association task and the probability of using word X to describe the meaning of word Y. Furthermore, we provide evidence that associative measures are altered in people suffering from Alzheimer's disease (AD) and predict their level of performance in a picture-naming task. We provide a parsimonious account of the experimental data gathered form these different sources of evidence according to the hypothesis that the links between a cue word and its associates can be viewed as binding a concept (the cue) to pieces of information regarding its meaning (the associates).

## Introduction

The word association technique first used by Galton (1879) consists of letting subjects respond to a cue word with the first word that comes to mind. After more than a century since this technique was first adopted, the nature of the relationship between a cue word and its associates is still a matter of debate. In the present work we propose (and provide empirical evidence in favor of) the hypothesis that the links between a cue word and its associates can be viewed as binding a concept (the cue) to pieces of information regarding its meaning (the associates).

A long standing neuropsychological tradition, which dates back to nineteenth century associationism, clearly refutes this view. This persistent skepticism about the role of language in shaping concepts' boundaries is shared by exponents of the two most influential approaches to semantic memory: the amodal approach and the embodied cognition approach (see Barsalou et al., 2003; Mahon and Caramazza, 2008, for critical reviews of these contrasting approaches). In keeping with the divergent assumptions underlying these approaches, the reasons for depriving language of semantic relevance are somewhat opposite in the amodal and embodied cognition factions. Sustainers of the former approach consider semantics as something represented in a much *more abstract* format than language, which in turn is considered one of several modality-specific sources of information about things in the world (see for example the hub-and-spoke model, Rogers et al., 2004; Patterson et al., 2007). By contrast sustainers of embodied cognition consider semantics as something much *more concrete* than language. In fact, in this view purely verbal tasks are independent from action and perception and semantic processing relies on the re-enactment of the same sensorimotor representations that become active during interaction with objects in the world (Barsalou, 1999).

In amodal approaches to semantic memory, the idea that associative links between words have nothing to do with semantics is clearly put forward by Fodor in his very influential essay on the modularity of mind (Fodor, 1983). In line with the 2 fold assumption that first the language recognition system is informationally encapsulated (i.e., it is insensitive to information specified outside of it) and second that semantic information is represented outside the language recognition system, Fodor argued in favor of the conclusion that although word recognition is known to be speeded up when words are placed in a coherent sentential context, semantic information is nevertheless not useful in guiding lexical access. In his view, if the sentential context, “I have the pepper but would you please pass the ___” speeds up response to the target word “salt” in a lexical decision (consisting of letting subjects decide if a letter string is or is not a real word), this occurs through the connections between the lexical nodes “pepper” and “salt” (the lexicon is of course represented within the language recognition system) and not because the language recognition system has access to the (semantic) knowledge that pepper and salt are currently used together to dress food. According to Fodor, if the language recognition system is able to behave as if it were using semantic knowledge, this is because “terms for things frequently connected in experience become themselves connected in the lexicon” (pp. 81–82). In other words, in the modular language recognition system lexical associations mimic semantic relationships outside this system, but (associative) relations between words are not primarily semantic.

Coming to the embodied cognition approach, Barsalou and colleagues are among the most explicit in considering word association a non-semantic phenomenon (Santos et al., 2011). According to these authors, lexical association reveals links between words but not between concepts because responding to a cue word with the first word that comes to mind is a task that does not rely on “situated simulation,” i.e., on the re-enactment of previous experiences with referents of the involved words (to respond “cat” to the cue word “dog” there is no need to imagine a scene involving cats and dogs). Since re-enactment of patterns of neural activity experienced during interaction with things in the world is the very basis of our semantic knowledge, the link between a cue word and its first associate cannot be semantic in nature.

This widespread disbelief that word association is a semantic phenomenon is reflected in the vast debate on the semantic vs. associative nature of word-word priming.

The paradigm of word-word priming is very similar to that of contextual facilitation discussed by Fodor (1983, see above). It consists of the reduction of reaction times in responding to a target word when it is preceded by a related prime word compared to an unrelated prime word. Thus, for example, deciding if a given target is a real word or a non-word (i.e., a lexical decision task) requires shorter reaction times if the target word “dog” is preceded by the prime word “cat” than by the unrelated word “chair.” An enduring issue is whether the priming effect observed in related pairs relies on their semantic or their lexical relationship.

Assuming, in keeping with Fodor (see above), that associative links rely on the co-occurence of words in texts, Lucas (2000) carried out a meta-analytic review of the priming literature to ascertain whether “there is a boost in priming from adding an associative relationship to an existing semantic one” (p. 619). This author found empirical support for this hypothesis; however, she concluded her review of the literature with the somewhat puzzling caveat: “Whether this associative boost is due to a particularly strong semantic relationship among associated words or to the addition of a co-occurrence relationship remains unclear” (p.627). Indeed, this statement sounds like an acknowledgment that word association might reveal the strength of semantic links rather than the presence of lexical relationships.

This argument was further developed by Hutchison (2003, see also Moss et al., 1995) who observed that most associated pairs are linked by an evident semantic relationship, but not necessarily one based on featural overlap; this holds for coordinate concepts (thus, for example, the dog concept is semantically related to the cat concept because both dogs and cats <have fur>, <are animals>, <live in homes> and so on) but also pairs like hammer and nail are clearly semantically related. Thus, although it is possible to find two members of a common semantic category that are not listed in associative norms (e.g., “pig” and “horse”), it is hardly possible to find associative pairs that do not have some kind of semantic relationship. As Hutchison (2003) reports, the vast majority of associated pairs listed in Palermo and Jenkins' (1964) norms are terms that are either characterized by featural overlap (like the following cue/associate pairs: synonymic, afraid/scared; superordinate, dog/animal; coordinate, sheep/goat) or by a concept-feature relationship (referring either to perceptual properties, e.g., canary/yellow, or functional ones, e.g., broom/sweep). By contrast, purely phrasal associates (baby/boy) represent a minority type.

In a recent paper (Zannino et al., 2015) we criticized the marginal role assigned to language in semantic memory by most current neuropsychological models and argued in favor of an alternative approach to conceptual knowledge that stresses the prominent role of words in shaping the boundaries of concepts. The present work focuses on the nature of associative links with the more general aim of acknowledging the role of language in conceptual knowledge. To this end, we attempt to provide empirical support for the hypothesis of the semantic nature of the links between associated words. In particular, as stated at the beginning of this introduction, we hold that words which are associated with a given cue represent bits of information that belong to the semantic representation of the cue word. It is well-known from associative norms that some cue words elicit the same few associates across all participants (These cue/associate pairs are said to have high associative strength) whereas other words elicit many associates, each of which is produced by only a few participants (These cue/associate pairs are said to have low associative strength). In fact, if we admit that to perform word association tasks participants can rely on either their idiosyncratic experience (a subject could respond “blood” to the cue word “glass” because he/she cut his/her finger with a broken glass the day before) or to more widely shared knowledge (probably at work in associations like “umbrella”/”rain”), then we will also be willing to believe that high dominant associates (i.e., responses produced by most participants to a given cue) rely on shared knowledge whereas low dominant associates rely on idiosyncratic personal experiences. Going a step further, we argue that the shared knowledge ground, where high dominant associates come from, is in fact the semantic knowledge shared by the speakers of a given natural language. In this view, non-idiosyncratic word associates represent pieces of semantic information about the cue word *and* cues having a high *associative agreement* (AAG), i.e., that elicit only a few associates with high associative strength, are likely to have a *core meaning* that is widely agreed upon among the speakers of a given natural language. Alternatively one could believe that participants in associative norms studies retrieve non-idiosyncratic associates from a common ground of lexical knowledge shared across the speakers of a given natural language. In this view, high associative agreement would rely on some lexical (as opposed to semantic) characteristics of the cue word. The lexical and semantic hypotheses about the nature of the associative links between words make several different predictions that we will attempt to empirically verify in the present study.

First, if, as we maintain, non-idiosyncratic associated words express semantic features of the cue, then the associative strength of a given cue/associate pair can be considered a measure of between subject agreement about the presence of a given feature in the meaning of a given cue word. Thus, based on our assumption there should be a relationship between associative strength and the presence of associated words in the definition of the cue word. Thus, for example, because the word pair “umbrella”/“rain” has high associative strength, the occurrence of “rain” should be high in the definitions of “umbrella” provided by a sample of subjects. To verify this prediction, we let healthy participants define concrete words for which we had previously collected associative norms and looked for a correlation between strength of the cue/associate link and frequency of occurrence of the associate in the cue word definitions produced by our subjects.

A second prediction regards the role of AAG in predicting performance on semantic tasks. We reasoned that if high AAG suggests the presence of a widely agreed upon core meaning it is arguable that it also predicts ease of processing of single words meanings. In tasks like picture naming a picture must be semantically interpreted in order to have access to the lexical label of the corresponding word (Humphreys et al., 1988; Alario et al., 2004). Thus, pictures corresponding to words with high AAG are expected to be easier to name than pictures corresponding to words with low AAG, particularly for subjects with reduced semantic resources. To verify this prediction, we enrolled a sample of subjects with Alzheimer's disease (AD) because the impairment of semantic memory is well documented in this population (see below). Participants with AD were asked to complete a picture naming task involving the same words for which AAG norms were previously collected in a sample of healthy subjects. Naming data were analyzed to search for a relationship between AAG and naming accuracy in the AD group.

Finally, if associative links are semantic in nature, it is expected that people with impaired semantic knowledge should exhibit altered measures of associative strength. To verify this hypothesis, the AD sample and a healthy control group were submitted to a free association task. Values of associative strength between cue and associated words were computed and compared across groups.

We chose to enroll AD patients to verify predictions two and three because there is a general agreement regarding the presence of a semantic impairment in AD (Hodges et al., 1991; Salmon et al., 1999; Chertkow et al., 2008; Cuetos et al., 2012; Zannino et al., 2015). It should be noted, however, that some authors maintain that in addition to the semantic deficit some degree of lexical impairment might also affect language performances in this population (Cuetos et al., 2012). This suggests caution in interpreting our findings. Nevertheless, as discussed below, we believe that the evidence gathered in this study provides strong support in favor of the hypothesis that associative links are semantic nature.

## Materials and methods

Norms of associative strength and AAG were collected in a set of 35 cue words. The same 35 items were then used to devise the three experiments included in this study: (i) written word definition, (ii) picture naming, and (iii) free association. This allowed us to verify the three predictions we argue are in keeping with the semantic nature of associative links: (i) the presence of a relationship between the associative strength of an associated word and its frequency of occurrence in the definition of the cue word; (ii) the ability of AAG to predict AD naming performance; and, (iii) the presence of altered associative links in AD.

### Collection of associative norms

#### Subjects

To obtain normative data we enrolled two samples of healthy subjects. The first sample comprised 20 participants (half male and half female, mean age = 32.7, SD = 14.44 range = 25–60 years). The second comprised 52 healthy subjects (26 females, 26 males) over 65 years of age (mean age = 72.9, SD = 7.01, range = 65–89) who were matched for age with the AD sample. The smaller subject sample (*n* = 20) was enrolled in a pilot study aimed at selecting the items on which normative data were then collected in the larger sample (*n* = 52).

#### Measures

In order to verify our predictions we needed two indices of associative relationships computed on the same 35 words that were included in the experimental tasks, i.e., a measure of associative strength related to cue/associated pairs and a measure of AAG related to single cue words. To quantify the relationship between cue and associate we used, as is customary, the Forward Associative Strength (FSG) measure, which expresses the proportion of subjects responding with a given associate to a given cue. The index regarding the associative agreement (AAG) of single cue words was devised for the purposes of this study. To compute it, we borrowed a metric, the *H* statistic, which is widely used to compute name agreement, i.e., the degree to which subjects are consistent in producing a word for a specific picture in a confrontation naming task. The 35-cue words set with corresponding *H*-index AAG is reported in Appendix. The *H* metric was first used by Snodgrass and Vanderwart (1980) for quantifying name agreement and is still the most widely used measure for this purpose (see for example Janssen et al., 2011). From a statistical point of view, the problem of quantifying cross-subject consistency in producing associates and the problem of measuring name agreement are superimposable; thus, we argued that recourse to a well-known metric for our new associative variable was preferable.

As is common practice, we computed *H* using the following equation:

$$\begin{array}{l} H=\overset{k}{\underset{i=1}{\sum }}{\text{p}}_{i}\;\log2\left(\frac{1}{pi}\right) \end{array}$$

where *k* is the number of different associates produced for a given cue word and *p* denotes the proportion of subjects producing a given associated word (i.e., the FSG for that cue/associate pair).

It should be stressed that apart from the fact that AAG applies to single cue words, whereas FSG applies to cue/associate pairs, the relationship between the two variables is very strong; in fact (see the above reported equation), in order to quantify AAG of a given cue word we used the FSG values of all the word pairs formed by that cue and its associates; moreover, the FSG between a cue and one of its associates expresses the degree of agreement on this pair among the subjects comprising the normative sample.

#### Item selection

The goal of the selection procedure was to find 35 concrete, easily depictable words that were likely to span from an intermediate to a high level of AAG in normative data collected in a group of 52 elderly Italian controls who were age matched to the AD sample enrolled in the experimental tasks (see section Norms Collection). This range of AAG was desirable in order to discard idiosyncratic associations and still obtain enough variability for our variable of interest. The selection procedure comprised two steps: a search among existing associative norms and a pilot study enrolling a small sample of 20 healthy subjects. In the first step, a large corpus of 107 concrete and easily depictable words was taken from existing associative norms by selecting those cues for which the cumulative FSG of the three most associated words was above 50% in one or more of the searched norms (This was done to discard cues with low AAG). Due to the scarcity of large normative databases for the Italian language, we also used norms collected for other European languages (Ferrand and Alario, 1998; Peressotti et al., 2002; Fernandez et al., 2004; Nelson et al., 2004; De Deyne and Storms, 2008). In a second step, the corpus of 107 words was administered for free association to a sample 20 subjects and FSG was computed for each cue word. Each participant performed the free association task individually in a single session. The subject was instructed to respond as fast as possible with the first word that came to mind. The experimenter read the cue word aloud and recorded the response. From the above described database we selected the 35 cue words to be used for the collection of associative norms in a larger sample of 52 healthy elderly subjects. To be included in the final set of cue words, items had to meet the following criteria: (i) in the pilot study, they had to have obtained a cumulative FSG > 50% on the three most associated words; (ii) they shared no associates with the other cue words included in the final set of 35 cues; (iii) none of the other 34 cue words were included among their associates; (iv) no words co-occurring with the cue in Italian proverbs or idiomatic expressions were included among their associates. Furthermore, in selecting the 35 final items, particular care was taken to select words whose pictorial representations were likely to produce very high name agreement; thus, words having currently used synonyms–e.g., “macchina”, “auto” “automobile” (car) were excluded.

#### Norms collection

The 35 selected cue words were administered to a sample of 52 healthy elderly subjects to quantify the relevant associative indices. Fifty-two different pseudo randomizations were carried out to present the 35 cue words in a different order to each subject and to avoid that words belonging to the same superordinate category occupied adjacent positions. Norms were collected individually using the same procedure adopted for the pilot study (see section Item Selection): the experimenter read the cue aloud and recorded the response; the subjects were instructed to say the first word that came to mind as fast as possible.

The collected data were used to compute the FSG between each cue-associate pair and the AAG of each single cue word using the *H* metric. The 35-cue word set with corresponding *H*-index AAG is reported in Appendix.

Seventeen of the 35 words in our corpus were also contained in Fernandez et al.'s (2004) norms. As cue words, this corpus contains the stimuli originally comprised in the normative data by Snodgrass and Vanderwart (1980); thus, the norms of Fernandez and colleagues, like ours, were collected on concrete words referring to depictable objects. Based on these analogies, we used the norms of Fernandez to tentatively compare the distribution of AAG in our corpus (selected so as to contain words with a relatively high level of AAG) with a large corpus of unselected concrete words. To this aim, we computed the *H*-index of AAG for all words comprised in Fernandez et al.'s (2004) norms and compared the distribution of AAG in the 17 words shared by the two corpora with the distribution of this variable in Fernandez et al.'s entire corpus. The mean *H*-index of the 17 words also included in our corpus was 3.6 (SD = 0.94, range 1.8–5.3); the mean of the same parameter computed on the whole corpus was = 4.6, SD = 0.92, and the range was 1.8–6.4. Considering that low levels of *H* indicate high agreement, we can say that our words spanned a large range of *H*-values but on average showed a relatively high AAG. In fact, the average *H*-index of the 17 words shared by the two corpora was significantly lower than that of Fernandez et al.'s whole corpus (*t* = 4.798, *df* = 16, *p* < 0.001). A direct comparison of the *H*-index computed on the two normative corpora was precluded by the fact that the number of participants was about 10 times higher in the norms of Fernandez; thus, idiosyncratic responses were more frequent in those norms and *H*-values were consequently higher. Consider that the *H*-index can range from 0 (maximum agreement) to infinity (maximum dispersion) and that its value increases with the increasing number of different responses given by the normative sample. Interestingly, however, the 18 (i.e., 35 min 17) words comprised in our corpus, which were not present in that of Fernandez, showed an average *H* computed on our norms which was very similar to that of the 17 words shared by both corpora. Thus, the considerations we made about the 17 common items can likely be extended to the entire 35-word corpus.

### Experimental investigation

#### Subjects

Three subject samples were submitted to the experimental investigations described next. The first sample, comprising 33 female and 30 male healthy adult subjects, carried out the written word definition task (mean age = 37.7, SD = 15.12; mean years of education = 14.6, SD = 2.99). A group of subjects suffering from AD and their matched controls participated in the picture naming and the free association task. The AD sample comprised 10 subjects attending the Brain Aging and Alzheimer's Disease unit of the IRCCS Santa Lucia Foundation in Rome. All of them met the revised clinical criteria established by the National Institute of Neurological and Communicative Disorders and Stroke-Association for probable AD (McKhann et al., 2011). Their medical history, neurological examination, brain imaging and laboratory tests provided assurance that their dementia symptoms could not be attributed to an illness other than AD. The control sample comprised 10 healthy, age and education matched subjects (NC). Demographic characteristics of the AD and NC samples and age and education adjusted scores on the Mini-Mental State Examination (Measso et al., 1993) are reported in Table 1. Age and years of education were not significantly different across groups (F, consistently <1) but, as expected, NC outperformed AD significantly on the Mini-Mental State Examination [*F*_(1, 18)_ = 46.6, *p* < 0.001].

**Table 1:** Demographic characteristics and MMSE scores of the experimental sample.

**Group**	**Sex (F/M)**	**Age**	**Education**	**MMSE**
AD	8/2	75.4 (6.60)	11.5 (4.14)	20.15 (3.64)
NC	7/3	74.7 (8.43)	12.3 (3.86)	28.65 (1.51)

*Sex frequencies, mean (standard deviation) for Age and Education level (expressed in years) and mean adjusted MMSE scores are reported for the different experimental groups*.

The protocol of this study was approved by the Independent Ethic Committee of the Santa Lucia Foundation (Comitato Etico Indipendente dell'IRCCS Fondazione Santa Lucia). All subjects gave written informed consent in accordance with the Declaration of Helsinki.

#### Tasks

##### Written word definition

The 35 cue words comprised in the free association task were administered to a group of 63 healthy subjects (see above) in a written definition task. The words to be defined were printed in a booklet of A4 sheets of paper, six to a page with two empty lines underneath. Ten different pseudo randomisations of the 35-word list were carried out with the same criteria used to collect the associative norms (see section Norms Collection) On the first page of the booklet the following instructions were printed: “write a brief definition (1–2 sentences) of the following words”. Booklets were given to the subjects who performed the task at home and returned it several days later.

Responses were scored according to the cue/associate pairs generated by the normative sample. A score was attributed to each cue/associate pair (e.g., camel/desert) that expressed the proportion of subjects who used the associate word to define the cue word. Thus, for example, the camel/desert pair received a score of 0.57 because 36 out of 63 subjects used “desert” to define “camel.”

##### Picture naming

This task and the one described in the next section were administered to the 10 AD subjects and their controls. Each subject carried out the two tasks in the same session in fixed order, beginning with the free association task and ending with the picture naming task. The pictures included in the naming task were the same 35 items referred to by the cue words in the free-association task plus another 35 concrete objects. Items not included in the association tasks were added with a double purpose: (i) to increase the statistical power of between groups comparisons, (ii) to mask the relationship between the free association and the naming task. We selected 70 colored photographs from the Internet to construct this task. Photographs depicted typical exemplars of the items to be named in a canonical view. Care was taken to select photographs with neutral backgrounds i.e., devoid of information relevant to the identity of the object to be named. Responses were scored as either correct or incorrect.

##### Free association task

Materials and procedure were the same as those adopted for collecting the normative data and already described in section Norms Collection. The scoring procedure was as follows: each response given by either an AD or a control subject was scored according to the FSG obtained in our norms, i.e., according to the proportion of subjects who gave the same response in the original normative task; e.g., the response “deserto” (desert) to the cue “cammello” (camel) was scored 0.27 because 14 out of the 52 subjects enrolled in the normative task responded this way. If a subject produced a response never given by the normative sample a score of 0 was given.

#### Analyses

Each of the three above-described experiments was aimed at providing support for a different prediction of our hypothesis regarding the semantic nature of associative links. The outcomes of the experimental tasks were submitted to a series of descriptive and inferential analyses. For each experiment we will describe next only those analyses considered critical for verifying the corresponding prediction. As for the written word definition task, the prediction to be confirmed was that of a relationship between FSG and usefulness of a given associate in defining the meaning of the corresponding cue word. To verify this, a Pearson correlation analysis was conducted between the FSG of the associated words that resulted from the norms collection (Section Norms Collection) and the frequency of occurrence of those same words in the definitions of the cue words. A significant correlation is the expected output based on our prediction. As for the picture naming task, the prediction to be confirmed was that of a predictive role of AAG on the naming accuracy of the AD group. To confirm this prediction, a logistic regression was carried out which had the naming responses of the AD subjects (coded as a dummy variable: 0 = wrong, 1 = correct) as dependent variable and AAG and word frequency (Bertinetto et al., 2005) of the to be named word as predictors. A significant contribution of AAG over and above a possible role of word frequency in predicting naming accuracy is considered to be in keeping with our prediction. Finally, as for the free association task we expected altered FSG in AD subjects as compared to NC. To verify this, we adopted a mixed model approach to demonstrate a group effect (AD vs. NC) over and above the predictive role of Subject and Word entered as random factors.

## Results

### Written word definition

As stated at the end of the introduction, the hypothesis of the semantic nature of associative links predicts that the FSG between a word pair should predict the frequency of occurrence of the associated word in the definition of the cue word. To verify this prediction we analyzed the output of our word definition task and searched for a relationship between the probability of producing a given word as a free associate to a given cue and the probability of producing the same word in the context of a written definition of the meaning of the cue word.

**Figure 1:**
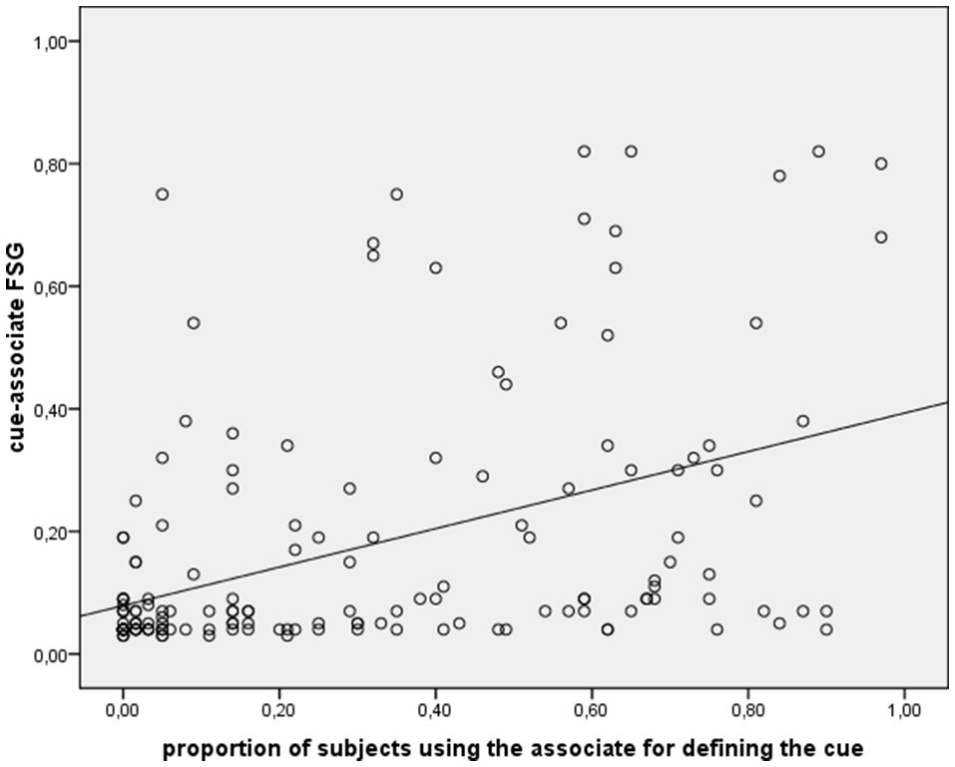
Scatterplot and last squares line showing the correlation between cue-associate FSG and proportion of subjects using the associate for defining the cue meaning.

Overall the normative sample produced 165 different, non-idiosyncratic (i.e., produced by at least two subjects) associates on the free association task. Across these 165 items, we performed Pearson's correlation coefficient between the proportion of subjects who produced a given item as a free associate and the proportion of subjects who produced the same item in the word definition task (throughout the paper, the Boostrap method was used to derive CI for correlations). This correlation (see Figure 1) turned out to be highly significant, *r* = 0.45, *p* < 0.001, 95% CI: 0.315–0.570, observed power (1-β) = 0.999. This result lends support to the hypothesis that subjects rely on their semantic representations in performing both tasks. It should be noted that the 52 healthy subjects in the normative data collection sample (see section Norms Collection) were older than the 63 subjects who performed the word definition task. Although this difference cannot be considered a serious confound (because noise resulting from the unmatched subject sample could only have loosened the critical correlation between our variables of interest not the opposite), we attempted to confirm this result by substituting the values obtained from the unmatched 52 subjects sample with those obtained from the 20 younger normal subjects enrolled in the pilot study (see section Subjects). This time age was comparable across groups [*F*_(1, 81)_ = 1.7, *p* = 0.189] As expected, the correlation between FSG and the reviewed values of AAG was still significant: *r* = 0.48, *p* < 0.001, 95% CI: 0.356–0.610, observed power (1-β) = 0.999.

### Picture naming

Our picture naming test comprised 70 items, 35 of which depicted items also comprised in the free-association and the written word definition task. As stated above (see section Item Selection), these items were selected to minimize the likelihood that correct unexpected answers would be produced in picture naming. To this end, words with currently used synonyms were excluded. Results of our naming task were in keeping with our expectations: only 5 and 4% of the correct naming responses given by the AD and NC subjects respectively did not coincide with the expected response. This value was considerably higher, i.e., 13%, for the 35 unselected items in the naming task.

**Figure 2:**
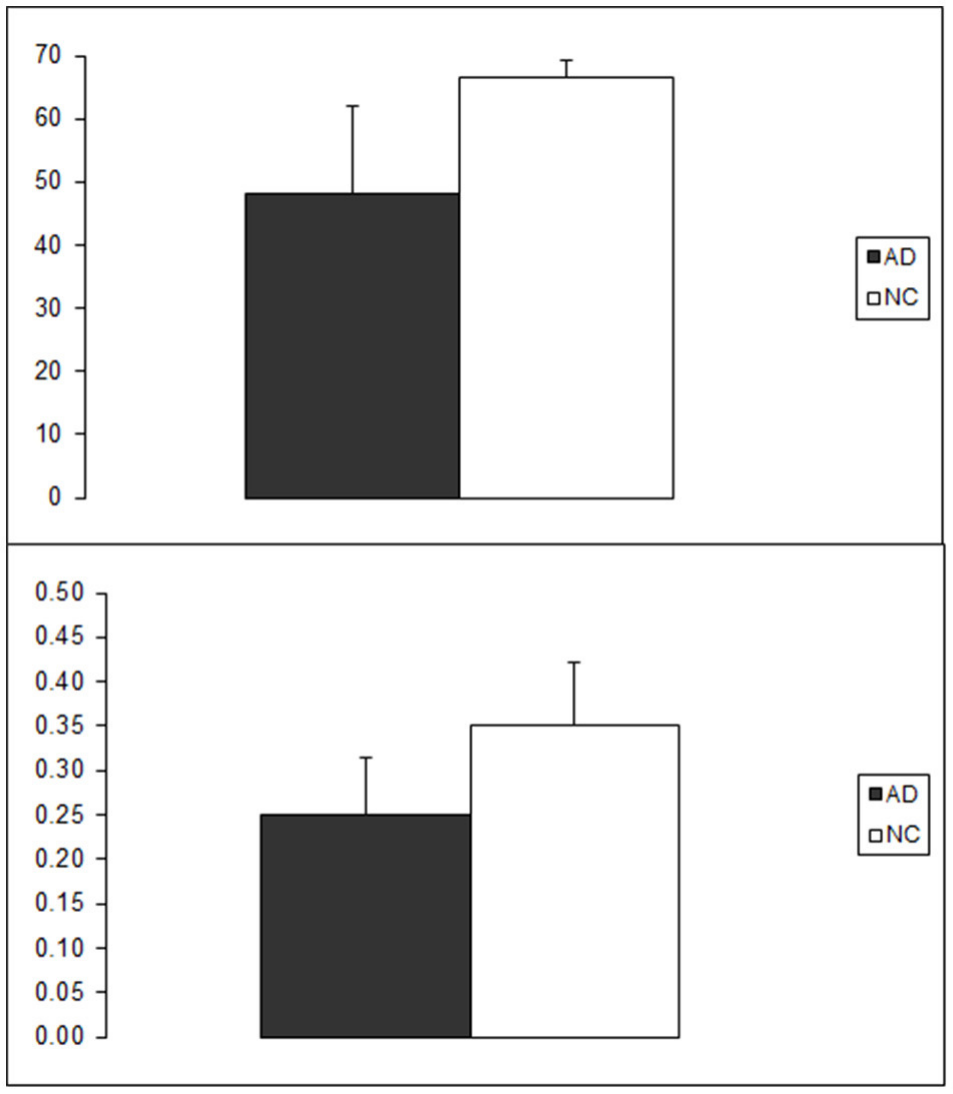
Group performance in the picture naming task (above) and in the Free association task (below). On the Y axis number of correctly named items and mean FSG are reported for the naming and free association task respectively. As can be seen, higher naming performance in the NC group is associated with the production of associates with higher FSG.

In the first analysis we wanted to verify whether our AD sample exhibited a naming impairment as compared to its matched control group. To this aim we compared naming accuracy on the corpus of 70 items across groups. As expected, NCs outperformed ADs. The mean number of correct responses (and SD) was 48.2 (13.9) and 66.5 (2.8) in the AD and the NC group, respectively (see Figure 2). AS the variance of the dependent variable was not equal across groups, a nonparametric analysis was carried out using the Mann-Whitney U-test. The cross-group difference in naming accuracy was statistically significant (U = 1, N_1_ = 10, N_2_ = 10; *p* < 0.001, two-tailed).

More interesting for our purposes was the investigation of the role of AAG in predicting the naming performance of the AD group. To this end we carried out a logistic regression analysis by entering as dependent variable each single naming response given by AD subjects coded as a binary variable (0 = wrong, 1 = correct) and as predictors we again entered word frequency and AAG (*H*-index) of the word to be produced. The full model significantly predicted naming accuracy (omnibus chi-square = 18.445, *df* = 2, *p* < 0.001) with both independent variables reliably predicting naming output: *H*-index, *B* = −0.469, Wald = 8.356, *p* = 0.004; word frequency, *B* = 0.012, Wald = 8.540, *p* = 0.003. Observed power (1-β) for frequency and H-index was 0.785 and 0.881, respectively. As the sign of the B coefficients revealed, words with high frequency and high AAG (i.e., low H-index values) were named better. It is widely agreed (see discussion) that word frequency predicts ease of processing at the lexical level; therefore, the fact that AAG reliably predicts naming accuracy while controlling for word frequency suggests that the two variables act at different processing levels. This impression is further confirmed by the lack of any significant correlation between the H-index and word frequency (*r* = 0.121, *p* = n.s.); if both were indices of ease of lexical processing, we should have found a correlation between them. In a further attempt to take apart the locus of word frequency and the AAG effect, we analyzed both variables as a function of error type. In particular, we performed two independent univarate ANOVAs to ascertain which kinds of errors showed a significantly different level of frequency/AAG as compared to the correct responses. Responses were categorized as correct responses (*n* = 247), semantic errors (*n* = 31; this category included coordinate errors—e.g., “horse” for “camel”—and superordinate errors-e.g., “transportation” for “sledge”), anomic errors (i.e., omissions, *n* = 45) and other errors (*n* = 27) including circumlocutions (e.g., “house for bees” for “hive”), non-correlated responses (e.g., “colors” for “puzzle”) and visual errors (e.g., “ball” for “olive”). Both H-index [*F*_(3, 346)_ = 3.2, *p* = 0.025] and frequency [*F*_(3, 346)_ = 4.2, *p* = 0.006] varied significantly across the different response types. More interestingly, concerning the H-index only semantic errors were characterized by a significantly different H-index compared to correct responses (correct responses = 2.35, semantic errors = 2.7, LSD test, *p* = 0.015). Importantly, items yielding semantic errors showed lower levels of AAG (i.e., higher values of H-index) compared to items yielding correct responses. Regarding word frequency, significant comparisons involved anomic errors (correct responses = 37.50 vs. anomic errors = 25.11, LSD test, *p* = 0.027) and other errors (correct responses = 37.50 vs. other errors = 16.52, LSD test, *p* = 0.003) whereas the frequency of words yielding semantic errors did not differ significantly from the frequency of words yielding correct responses. Also in this case, differences across response types were in the expected direction, i.e., lower frequency for incorrectly named words. We interpret these findings as suggesting that the two psycholinguistic variables have different action levels (see Discussion).

### Free association task

The mean FSG (and SD) of the associated words produced by AD and NC participants was 0.25 (0.065) and 0.35 (0.071), respectively (see Figure 2). We were interested in demonstrating that this difference was statistically reliable taking into account the potential effect of inter-subject and inter-item uncontrolled variability. To this end the data obtained in the free association tasks were analyzed with a mixed effects model approach. FSG of any particular cue/associate pair produced by any particular participant was entered as dependent variable, Group (AD vs. NC) and H-index were entered as fixed factors and Subject (i.e., participant 1–20) and test Item (i.e., word 1–35) were entered as random factors. Although the effect of AAG was not relevant to verify our prediction (i.e., altered FSG in the AD group), entering H-index in the model was warranted because the 35 cue words were selected in order to span a wide range of AAG values and it is expected that AAG of a cue affects FSG of its associated (see equation in section Measures). Bayesian information criterion (BIC) indicated that removing the Item random factor increased the model fit (BIC = 12.5 and 6.0 with and without the Item factor respectively). Thus, the simpler model will be described next. Both the Group [*F*_(1, 245)_ = 7, *p* = 0.009] and the H-index [*F*_(1, 637)_ = 385, *p* < 0.001] effects turned out to reliably predict FSG whereas the Group by H-index interaction did not reach statistical significance [*F*_(1, 637)_ = 1.9, *p* = 0.159]. Subject entered as random effect also contributed to the model fit (*Z* = 2.033; *p* = 0.042).

This results confirm the prediction that FSG is altered in the AD group. In particular, FSG appear to be reduced in the pathological population. Furthermore, the lack of a significant Group by H-Index interaction suggests that this reduction is comparable across words spanning a large range of AAG values.

In order to be sure that the observed cross groups difference in FSG was not due to the well-known trend in AD subjects to produce high frequency words (Cuetos et al., 2012), in another item analysis we compared the mean production frequency (Bertinetto et al., 2005) of the associates across groups. The mean frequency (number of occurrences per 3 million) of the associates produced for the 35 cue words was comparable in the AD group (mean 302.2, SD = 227.3) and the NC group (mean = 339.6, SD = 542.9), *t* = 0.469, *df* = 34, *p* = n.s. This result rules out the possibility that the FSG effect was the epiphenomenon of a Frequency effect.

## Discussion

The present work is an attempt to provide empirical support for the hypothesis that the relationships between a cue word and its free associates are semantic in nature. This counters the prevailing view that associative links reveal lexical relationships between words. To do this we devised two experiments involving semantic tasks and investigated the effects of word association indices on the performance of healthy participants and AD subjects who are supposed to suffer from a disruption of semantic knowledge. The first experiment consisted of a word definition task that was carried out by cognitively intact individuals. The second experiment consisted of a picture naming task that was performed by a sample of AD subjects and their normal controls. Finally, in a third experiment, involving AD subjects, we investigated the effect of impaired semantic memory in a free association task.

The three experiments provided results that consistently support the semantic nature of associative links.

The evidence gathered from the first experiment regards the relationship between FSG of a cue/associate pair and the proportion of healthy subjects who use that associate to define the meaning of the cue word. We found that the proportion of subjects who use word X to define word Y increases with increasing FSG between word Y (cue) and word X (associate). In our view this strongly suggests that FSG captures the usefulness of word X in defining word Y. Moreover, it is likely that words which are more useful in defining a given meaning carry important bits of semantic information about that meaning. Thus, FSG likely expresses the semantic relevance of the associates for the meaning of the cue word. This clearly hints at the semantic nature of associative links.

The main finding of the second experiment regards the predictive role of AAG in the naming ability of individuals with AD. Assuming that people with AD fail on naming tasks because of their disrupted semantic memory, the finding of a relationship between AAG and naming performance in this population argues strongly in favor of the semantic nature of associative links. Regarding the mechanisms through which AAG might influence naming performance, we argue as follows: Words with high AAG elicit only few associates in a normative subject sample. In our view, (non-idiosyncratic) associates represent pieces of semantic information (i.e., semantic features); thus, if most subjects select the same few associates for a given cue word, this means that there is high agreement about the most useful features for describing the meaning of that word. Thus, high AAG entails high semantic agreement and meanings upon which subjects strongly agree are likely to be more resilient against the pathological disruption of conceptual knowledge that occurs in AD. Although there are many hints of the semantic nature of naming errors in AD^1^, some authors have argued that there is also a lexical deficit in AD (Cuetos et al., 2012). Thus, the effect of a putative lexical impairment on the naming performance of our AD sample cannot be definitely ruled out. Therefore, at variance with our view one could hypothesize that AAG captures some *lexical* characteristics of the cue word and exerts its effect on AD naming through mechanisms different from the semantic one we proposed above. There are two related findings, however, which in our opinion run counter to this possibility. First, in the word sample included in our experiment, AAG did not correlate with word frequency and, second, AAG predicts naming accuracy over and above the effect of word frequency. Because this latter index is considered to be firmly associated with the lexical, post semantic step of word production (see Nozari et al., 2010 for an in-depth discussion of functional locus of the frequency effect) based on the assumption that AAG also measures the ease of lexical processing of a given word, we should expect some degree of correlation between word frequency and AAG. Similarly, assuming that both AAG and word frequency exert their effect at the lexical level of processing, an independent contribution of both variables in predicting naming accuracy would not be expected. Further evidence arguing in favor of a semantic locus of the AAG effect on AD naming performance was obtained by taking error type into account. In the AD sample AAG turned out to be significantly lower in items that give rise to semantic naming errors than in items that generated correct responses. By contrast, no significant differences in AAG levels were found when correct responses were compared with anomic errors or other errors. By contrast, low word frequency characterized anomic errors and other errors as compared to correct responses, while words giving rise to semantic errors did not show lower frequency as compared to correctly named words. The finding that the two variables are associated with different errors types clearly suggests that they act at different functional loci. More importantly for our purposes, the error type reliably associated with AAG (i.e., coordinate and superordinate errors) “has been taken as (…) evidence that one of the major cognitive impairments suffered by AD patients is a disintegration of their conceptual semantic system” (Cuetos et al., 2012, p.245).

The main result of the last experiment of the present study was that FSG is reduced in AD; i.e., subjects with AD are less consistent than NCs in producing associates to a given cue. As we indicated above, the assumption of a pure semantic deficit in AD is not universally agreed upon and thus the finding of reduced FSG in this population cannot be unequivocally interpreted because it could arise from the loosening of links between semantic features (according to the hypothesis of the semantic nature of associative links) as well as from disruption at the lexical processing level (in keeping with the alternative hypothesis of the lexical nature of associative links).

Nevertheless, a secondary finding of the same experiment counters the alternative lexical hypothesis. Indeed, the AD subjects produced associated words in our free association task with virtually the same frequency as NCs. On the assumption that FSG is altered in AD due to a lexical impairment, we should have found increased word frequency of associated words produced by AD because high frequency words are easier to process at this functional level.

There is wide agreement among neuropsychologists that associative links are lexical and non-semantic in nature *because* they rely on the co-occurrence of words in texts. In fact, this conclusion rests on the assumption that co-occurrence patterns of words in text cannot contribute to the definition of concept boundaries (*because* concepts exist independently from the lexical labels that refer to them). Sustainers of the non-semantic nature of word associations are right in affirming a relationship between the distribution of words in printed and spoken text and associative links. This relationship is in fact demonstrated by the “correlation between associative strength and co-occurrence of words in large language corpora” (Moss et al., 1995, p. 864). However, starting from other premises, the finding of this correlation favors the opposite conclusion, i.e., that associative links *are* semantic in nature *because* semantics rely on the relations between words in text. There is wide agreement regarding this latter point of view outside the neuropsychological tradition. In linguistics, for example, the “contextual approach” to semantics assumes that “the semantic properties of a lexical item are fully reflected in appropriate aspects of the relations it contracts with actual and potential contexts” (Cruse, 1986, p.1). Psycholinguistic approaches based on statistical computations applied to large text corpora also share the common tenet that meanings arise from relationships between words (e.g., see the Latent Semantic Analysis approach, Landauer et al., 1998, see Bruni et al., 2014 for recent developments in this approach).

Interestingly, in order to prove their semantic relevance, sustainers of statistical measures have successfully used these variables to predict priming effects (see for example Jones et al., 2006) as well as other purported semantic phenomena in humans (e.g., synonym judgment, Landauer et al., 1998). Noteworthy, as we mention in the Introduction, priming effects have also been interpreted as arising from more shallow relations between prime and target, namely, lexical ones. In fact, if we admit, in keeping with Fodor (1983), that relations between words can mimic relations between things in the world, it becomes impossible to argue in favor of the truly semantic nature of word relations from their ability to predict human performance on semantic tasks. What Fodor said about associative links could also apply to any kind of statistical relationship between words: they simply “are the means whereby stupid processing systems manage to behave as though they were smart ones” that is, as if they were handling semantic information (1983, p. 81).

The interpretation of the evidence provided by the present work suffers from the same ambiguity: apart from the potential confound of a putative lexical deficit in AD subjects, it is impossible to definitely rule out that associative links simply mimic, at the lexical level, semantic relations which hold in a “smarter” amodal or embodied system.

Nevertheless, the present results represent a case for language oriented approaches in so far as they easily and straightforwardly fit the predictions of a hypothesis (i.e., the semantic nature of associative links) which gives language a fundamental role in constraining the organization of conceptual knowledge in humans. In fact, summarizing our results we can say that for several reasons the semantic hypothesis of associative links seems preferable to the alternative lexical hypothesis. First, it is more parsimonious because it can account for the results of all of our experiments, whereas the alternative explanation is unsuited to account for the findings of experiment 1. Second, the semantic hypothesis provides a principled account of the results of experiment 2, particularly concerning the pattern of modulatory effects different error types exert on the action of AAG and word frequency on naming accuracy. Third, the independence of AAG and word frequency (a variable exerting its effect at the lexical level) that emerged from the findings of experiments two and three suggests that the two variables influence different cognitive processes.

## Author contributions

All authors contributed to the conception of the work, and to the interpretation of the data, approved the final version of the manuscript and agreed to be accountable for all aspects of the work and qualify for authorship GDZ designed the experiments, performed the analyses and wrote the manuscript. RP selected and diagnosed the pathological sample AT selected the control sample and collected the data CC revised the manuscript GC revised the manuscript.

### Conflict of interest statement

The authors declare that the research was conducted in the absence of any commercial or financial relationships that could be construed as a potential conflict of interest.

## Footnotes

^1^Although a review of the literature on this topic is beyond the scope of this study, we would like to mention two of the most often reported findings supporting this argument. First, AD patients fail on the same items they are unable to name across a range of different semantic tasks that do not require naming responses (Salmon et al., 1999, p. 110). Second, as compared to elderly controls, the qualitative analysis of naming errors in AD revealed a preponderance of superordinate and semantic associative errors (indicating a semantic genesis) and a scarcity of circumlocutory responses (indicating lexical damage) (Hodges et al., 1991, p.1555).

## Appendix

**Table:**

**CUE**	**ENGLISH TRANSLATION**	***H-*INDEX OF AAG**
ALVEARE	HIVE	1.05
CAMMELLO^*^	CAMEL	2.95
CANGURO^*^	KANGAROO	3.03
CARROZZA	CARRIAGE	1.87
CLESSIDRA	HOURGLASS	1.85
COMPASSO	COMPASSES	2.88
DITALE^*^	THIMBLE	2.20
ELICOTTERO^*^	HELICOPTER	3.57
FRIGORIFERO^*^	REFRIGERATOR	3.30
GHIANDA	ACORN	2.49
GIRAFFA^*^	GIRAFFE	3.10
INCENSO	INCENSE	2.88
INTERRUTTORE^*^	SWITCH	2.05
LATTINA	CAN	3.04
OCCHIALI	GLASSES	1.88
OLIVA	OLIVE	1.81
OMBRELLO^*^	UMBRELLA	1.11
PECORA^*^	SHEEP	3.48
PETTINE^*^	COMB	1.45
POLPO	OCTOPUS	2.73
POMODORO^*^	TOMATO	3.45
PORTAFOGLIO	WALLET	1.73
PUZZLE	PUZZLE	3.45
RAGNO^*^	SPIDER	2.52
RINOCERONTE^*^	RHINOCEROS	2.91
RUBINETTO	FAUCET	1.18
SERRATURA	LOCK	1.77
SIGARETTA	CIGARETTE	1.69
SLITTA^*^	SLED	2.65
TAZZA^*^	CUP	2.92
TERMOMETRO	THERMOMETER	1.49
VALIGIA^*^	SUITCASE	2.86
VAMPIRO	VAMPIRE	3.71
VASO^*^	VASE	1.18
VULCANO	VOLCANO	2.93

* *Word present in Fernandez et al. (2004)*.
